# A novel role for vaping in mitochondrial gene dysregulation and inflammation fundamental to disease development

**DOI:** 10.1038/s41598-021-01965-1

**Published:** 2021-11-23

**Authors:** Stella Tommasi, Niccolo Pabustan, Meng Li, Yibu Chen, Kimberly D. Siegmund, Ahmad Besaratinia

**Affiliations:** 1 Department of Population and Public Health Sciences, USC Keck School of Medicine, University of Southern California, M/C 9603, Los Angeles, CA 90033 USA; 2 USC Libraries Bioinformatics Service, University of Southern California, NML 203, M/C 9130, Los Angeles, CA 90089 USA

**Keywords:** Genetics, Molecular biology, Biomarkers, Risk factors, Pathogenesis, Inflammation

## Abstract

We constructed and analyzed the whole transcriptome in leukocytes of healthy adult vapers (with/without a history of smoking), ‘exclusive’ cigarette smokers, and controls (non-users of any tobacco products). Furthermore, we performed single-gene validation of expression data, and biochemical validation of vaping/smoking status by plasma cotinine measurement. Computational modeling, combining primary analysis (age- and sex-adjusted limmaVoom) and sensitivity analysis (cumulative e-liquid- and pack-year modeling), revealed that *‘current’* vaping, but not *‘past’* smoking, is significantly associated with gene dysregulation in vapers. Comparative analysis of the gene networks and canonical pathways dysregulated in vapers and smokers showed strikingly similar patterns in the two groups, although the extent of transcriptomic changes was more pronounced in smokers than vapers. Of significance is the preferential targeting of mitochondrial genes in both vapers and smokers, concurrent with impaired functional networks, which drive mitochondrial DNA-related disorders. Equally significant is the dysregulation of immune response genes in vapers and smokers, modulated by upstream cytokines, including members of the interleukin and interferon family, which play a crucial role in inflammation. Our findings accord with the growing evidence on the central role of mitochondria as signaling organelles involved in immunity and inflammatory response, which are fundamental to disease development.

## Introduction

Electronic cigarettes (e-cigs) are battery-operated vaporizers that simulate combustible tobacco cigarettes^1–3^. E-cigs heat a liquid and convert it into an inhalable vapor for users^4^. The liquid (e-liquid/e-juice) is a mixture of solvents (*e.g.*, propylene glycol and glycerin), flavorings, and varying concentrations of nicotine, although e-liquid with no nicotine is also available^5,6^. E-cig use is often referred to as *‘vaping’*, and e-cig users are known as ‘*vapers*’^4^. Chemical analyses of e-cig vapor have revealed the presence of some of the same toxicants and carcinogens as those found in cigarette smoke, including carbonyl compounds, volatile organic compounds, free radicals, and heavy metals, although generally at much lower levels^2,3,7^. The reduced concentrations of these chemicals in e-cig vapor are consistent with the fact that e-cigs, unlike conventional cigarettes, do not *‘burn’* tobacco to produce inhalable materials^2,7,8^. This has led to the perception that e-cig use/vaping is safe or less-harmful than cigarette smoking^9,10^. Whereas the reduced levels of toxicants and carcinogens in e-cig vapor may imply risk mitigation, they do not, however, indicate risk elimination^2,8,11^. In fact, exposure to many constituents of e-cig vapor, at various concentrations, has been associated with a variety of cardiovascular-, immune-related (inflammatory), and respiratory diseases, and cancer^1,5,7,8,12,13^. Currently, e-cig use is widespread among adolescent never-smokers and adult smokers seeking a less-harmful alternative to regular cigarettes^6,9,14^. Notwithstanding the popularity of vaping, the long-term health consequences of e-cig use are largely unknown^2,4,7^.

Adult e-cig users are likely to co-use e-cigs and combustible cigarettes (*i.e.*, dual users) or have a prior history of smoking (*i.e.*, ex-smokers)^9,14^. To investigate the biological effects of e-cig use in adults, it is, therefore, important to tease out the consequences of vaping while accounting for the confounding effects of smoking. The existing literature on the ‘potential’ health risks of vaping is often criticized by the fact that study subjects in many reports consist of adult e-cig users with current or past smoking habits, *i.e.*, dual users or vapers ex-smokers, respectively^4^. This has complicated the interpretation of the results as it is unclear whether the observed effects in e-cig users are due to: (I) persistent effects of past smoking (in former smokers) or current smoking (in dual users); (II) current vaping only; or (III) a combination of the two factors^4,9^. The present study aims to disentangle the biological effects of vaping in adult e-cig users while accounting for smoking as a potential confounder.

Recently, we have constructed the oral transcriptome in adult e-cig users and cigarette smokers as compared to non-users (non-vapers nonsmokers) by RNA-sequencing (RNA-seq) analysis^15^. We have demonstrated that vapers, similarly to smokers, display significant dysregulation of functionally important genes in oral epithelial cells, a target cell type for tobacco-related diseases^15^. The dysregulated genes and functional pathways in vapers were partly similar to, but mostly distinct from those of smokers^15^. These findings accord with the known similarities and differences in chemical composition of e-cig vapor and cigarette smoke^1,2,8^. In a follow up study, we have also shown key epigenetic changes, including hypomethylation of repetitive DNA elements and global loss of DNA hydroxymethylation, which are hallmarks of cancer and other chronic diseases^16–18^, in peripheral blood leukocytes of vapers and smokers as compared to nonsmokers^12^. Building on these findings, we have utilized RNA-seq technology in combination with bioinformatic approaches and computational modeling to segregate the biological consequences of vaping from smoking in healthy adult vapers (with and without a history of smoking) and ‘exclusive’ cigarette smokers. More specifically, we have investigated the global expression of genes and modulation of functional pathways and gene networks in peripheral blood leukocytes of vapers and smokers in comparison to control nonsmokers non-vapers (*n* = 37, 22, 23, respectively).

Using a two-pronged approach, we have first performed age- and sex-adjusted limmaVoom^19^ for differential gene expression analysis^20–22^. Subsequently, we have used ordinal sensitivity analysis^23^ to seek the association between vapers’ differentially expressed genes (DEGs) and the intensity and duration of past smoking (calculated as ‘pack year’). To confirm the consistency and robustness of our analysis, we have also performed analogous sensitivity analyses to examine the dependence of DEGs in vapers and smokers on vaping and/or smoking indices (*i.e.*, cumulative e-liquid (cum e-liq) consumption (ml) and pack year (PY)). Furthermore, we have performed single-gene validation of the expression data by reverse transcription quantitative polymerase chain reaction (RT-qPCR)^12^, and biochemical validation of vaping/smoking status by measuring plasma cotinine levels in the study subjects ^5^, using an enzyme-linked immunosorbent assay (ELISA)^15^.

## Results

### Genome-wide differential gene expression analysis: primary model

**Figure 1:**
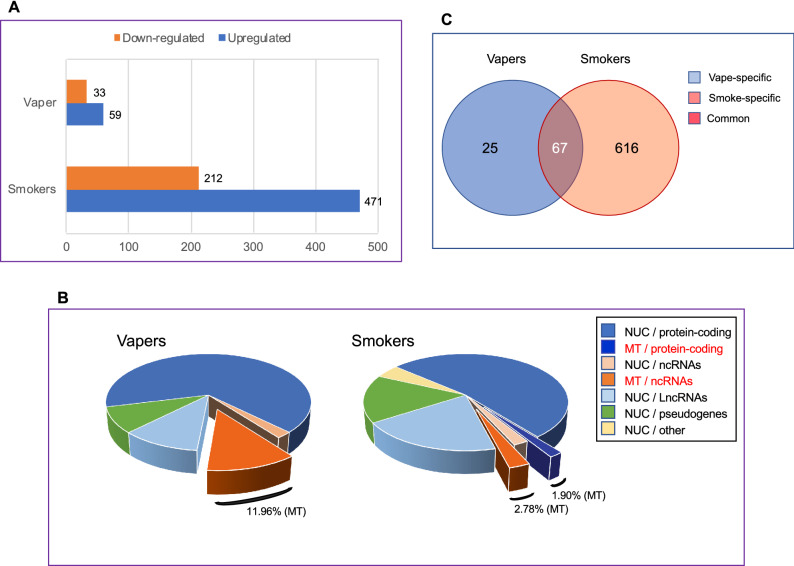
Differential expression of genes detected by RNA-seq in vapers and smokers as compared to controls. **(A)** Numbers of up-regulated and down-regulated genes in vapers and smokers are indicated (FC > 1.5 and FDR < 0.1) **(B)** Gene/transcript biotypes (based on Ensembl classification) in vapers and smokers. Percentages of mitochondrial DEGs (protein-coding and noncoding) in vapers and smokers are specified. NUC, nuclear; MT, mitochondrial. **(C)** Venn diagram of differentially expressed genes (DEGs) in vapers and smokers.

To evaluate the influence of vaping *vs*. smoking on global gene expression, we used the limmaVoom with quality weights framework^19^ to detect differential expression of genes in leukocytes of current vapers and smokers as compared to controls, while adjusting for age and sex as covariates. As shown in Fig. 1A, both vapers and smokers had substantial numbers of DEGs; however, the number of DEGs in smokers was ~ 7.4 times higher than that in vapers (683 *vs*. 92; > 1.5 fold-change and FDR < 0.1). The DEGs in vapers consisted of 59 up-regulated (64.1%) and 33 down-regulated (35.9%) genes (Fig. 1A). In smokers, there were 471 up-regulated (69.0%) and 212 down-regulated (31.0%) genes (Fig. 1A). Detailed lists of the DEGs in vapers and smokers, along with other relevant information, are provided in Supplementary Tables S1 and S2, respectively.

Of the 92 DEGs identified in vapers, 61 are protein-coding genes (66.3%), while the remaining 31 DEGs (33.7%) transcribe for regulatory non-coding RNAs (both short- and long non-coding RNAs) and non-functional elements, including pseudogenes (based on the GENCODE & Ensembl gene/transcript biotype classification) (Fig. 1B). In smokers, 374 of the 683 DEGs (54.8%) are protein-coding, while the remaining 309 DEGs (45.2%) belong to several classes of gene/transcript biotypes, including short non-coding RNAs (ncRNAs) (4.8%), long-non-coding RNAs (lncRNAs) (19.9%), pseudogenes (16.0%), and other elements of unknown function (4.5%) (Fig. 1B). Collectively, vapers, as compared to smokers, have an over-representation of protein-coding DEGs and an under-representation of non-protein-coding DEGs (*P* = 0.047; Yates corrected Chi-square test). Of significance, 12.0% of DEGs in vapers (11 out of 92) are of mitochondrial origin, including one DEG that encodes mitochondrial ribosomal RNA (MT-rRNA) and 10 DEGs that code for mitochondrial transfer RNAs (MT-tRNAs) (both classified as ncRNA sub-types). In smokers, however, 4.7% of DEGs (32 out of 683) consist of mitochondrial genes, which include 13 protein-coding genes, two MT-rRNAs, and 17 MT-tRNAs. Of these, nine are common to vapers, including one MT-rRNA and eight MT-tRNAs. Although the proportion of mitochondrial DEGs in smokers is smaller than that in vapers, the absolute number of dysregulated mitochondrial genes in smokers (32) is remarkable considering that the mitochondrial genome consists of only 37 genes^24^. Of these, 13 genes are protein-coding and the remaining 24, including two MT-rRNAs and 22 MT-tRNAs, are used for the translation of those 13 polypeptides^24^. Notably, all the dysregulated mitochondrial genes (protein-coding and non-coding) in both vapers and smokers are over-expressed (Supplementary Tables S1 and S2).

Figure 1C is a Venn diagram of the DEGs identified in vapers and smokers as compared to controls. Overall, there are three categories of DEGs in vapers and smokers as compared to controls: (1) vape-specific DEGs: genes differentially expressed in vapers only; (2) smoke-specific DEGs: genes differentially expressed in smokers only; and (3) common DEGs: genes differentially expressed in both vapers and smokers. The vape-specific DEGs account for 27.2% of all DEGs in vapers (25 out of 92 DEGs), whereas the smoke-specific DEGs comprise 90.2% of all DEGs in smokers (616 out of 683 DEGs). The common DEGs comprise 72.8% and 9.8% of all DEGs in vapers and smokers, respectively (Fig. 1C). Thus, the overwhelming majority of DEGs in vapers are common to those found in smokers. This implies that the identified DEGs in vapers, especially those common to vapers and smokers (category 3 from above), are likely due to: (I) exposure to similar chemical(s) present in both e-cig vapor and cigarette smoke; and/or (II) persistent effects of past smoking in vapers who have a history of smoking. To examine these possibilities, we have used ordinal sensitivity analysis to seek the association between DEGs in vapers (with and without a history of smoking) and indicators of *‘current’* vaping and *‘past’* smoking (cum e-liq and PY, respectively). In addition, to test the dependence of DEGs in vapers and smokers on vaping and/or smoking intensity and duration (*i.e*., dose), we performed sensitivity analysis for cum e-liq and PY in vapers, and for PY in smokers (*see*, below).

### Ordinal sensitivity analysis for differential gene expression: sensitivity model

Of the 92 DEGs in vapers identified by primary analysis, 75 (81.5%) showed concordant expression results when examined by cum e-liq sensitivity analysis (Supplementary Table S1). In contrast, none of the DEGs in vapers characterized by primary analysis, yielded differential expression results when tested by PY sensitivity analysis. Fifty three out of 67 (79.1%) DEGs common to vapers and smokers under the primary model, were consistently differentially expressed in vapers when tested by cum e-liq sensitivity analysis. Likewise, 22 out of the 25 (88.0%) vape-specific DEGs under the primary model, exhibited consistent differential expression results in vapers when analyzed by cum e-liq sensitivity model. In all cases, the DEGs in vapers, which showed concordant expression results under the two models, yielded stronger associations with vaping index in cum e-liq sensitivity analysis than in primary analysis (Supplementary Table S1). This was reflected by the smaller adjusted *P*-value (FDR) for each concordant DEG in the cum e-liq sensitivity analysis than the corresponding values in primary analysis. Furthermore, in vapers, the ‘*combined*’ adjusted *P*-values for all DEGs, vape-specific DEGs, and common DEGs, which showed concordant expression results under the primary and cum e-liq sensitivity models, were smaller under the latter model (Supplementary Table S1). In each case, the ‘*combined*’ adjusted *P*-value was calculated by averaging the *P*-values of all genes showing concordant statistically significant results in the primary and sensitivity analyses. Together, these data indicate that the vast majority of DEGs in vapers (79.1 to 88.0%) are dose-dependently associated with vaping index (cum e-liq) but not with past smoking index (PY).

**Figure 2:**
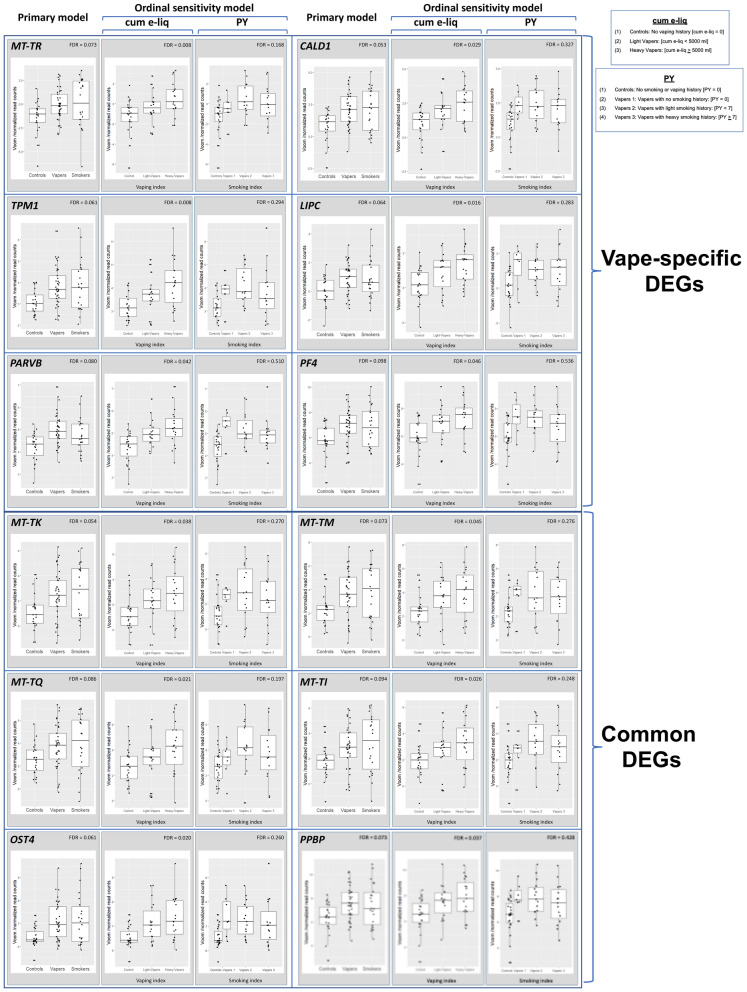
Visualization of the results of primary and ordinal sensitivity analyses—Vaping. Gene expression results for six vape-specific DEGs (upper panel) and six common DEGs (lower panel), as determined by primary and cum e-liq- and PY sensitivity analyses, are shown. Concordant statistically significant differential expression results for target genes in primary and cum e-liq sensitivity analyses, but not in PY sensitivity analysis, indicates that e-cig use, but not past smoking, is significantly associated with gene dysregulation in vapers. In the cum e-liq model, vapers were divided in two categories, including Light vapers [cum e-liq < 5000 ml], and Heavy vapers [cum e-liq ≥ 5000 ml], with Controls who had no vaping history. In the PY sensitivity model, vapers were stratified into three categories, including Vaper 1: No smoking history [PY = 0]; Vaper 2: Light smoking history [PY < 7]; and Vaper 3: Heavy smoking history [PY ≥ 7], with Controls who had no smoking or vaping history. Distribution of data within each group is shown by a combination of scatter plots (to display individual values) and box and whisker plots (to highlight the minimum, first quartile, median, third quartile, and maximum values as well as outlier(s) (if any)). In the scatter plots, identical values are overlaid and presented as a single circle (‘°’). In the box and whisker plots, the *‘lower’* and *‘upper’* edges of boxes represent the 1st and 3rd quartiles, respectively (25 and 75 percentiles, resp.). Horizontal lines within the boxes represent the medians (2nd quartile or 50 percentile). The *‘lower’* and *‘upper’* vertical lines extending from the boxes, also known as “whiskers”, represent the lowest and highest data points, respectively, excluding any outliers (minimum and maximum values, resp.).

To further highlight the dependence of DEGs in vapers on cumulative e-cig exposure, but not on intensity and duration of past smoking (PY), we have visualized the results of primary and cum e-liq- and PY sensitivity analyses for several randomly selected target genes. Figure 2 shows the visualization results for six vape-specific DEGs (upper panel) and six common DEGs (lower panel) in vapers *vs*. controls, as determined by primary and cum e-liq- and PY sensitivity analyses. In all cases, the target genes showed concordant statistically significant differential expression results in primary and cum e-liq sensitivity analyses, but not in PY sensitivity analysis. The associations between differential expression of the target genes and vaping index were stronger in cum e-liq sensitivity analysis than in primary analysis, as reflected by the lower FDR (Fig. 2).

**Figure 3:**
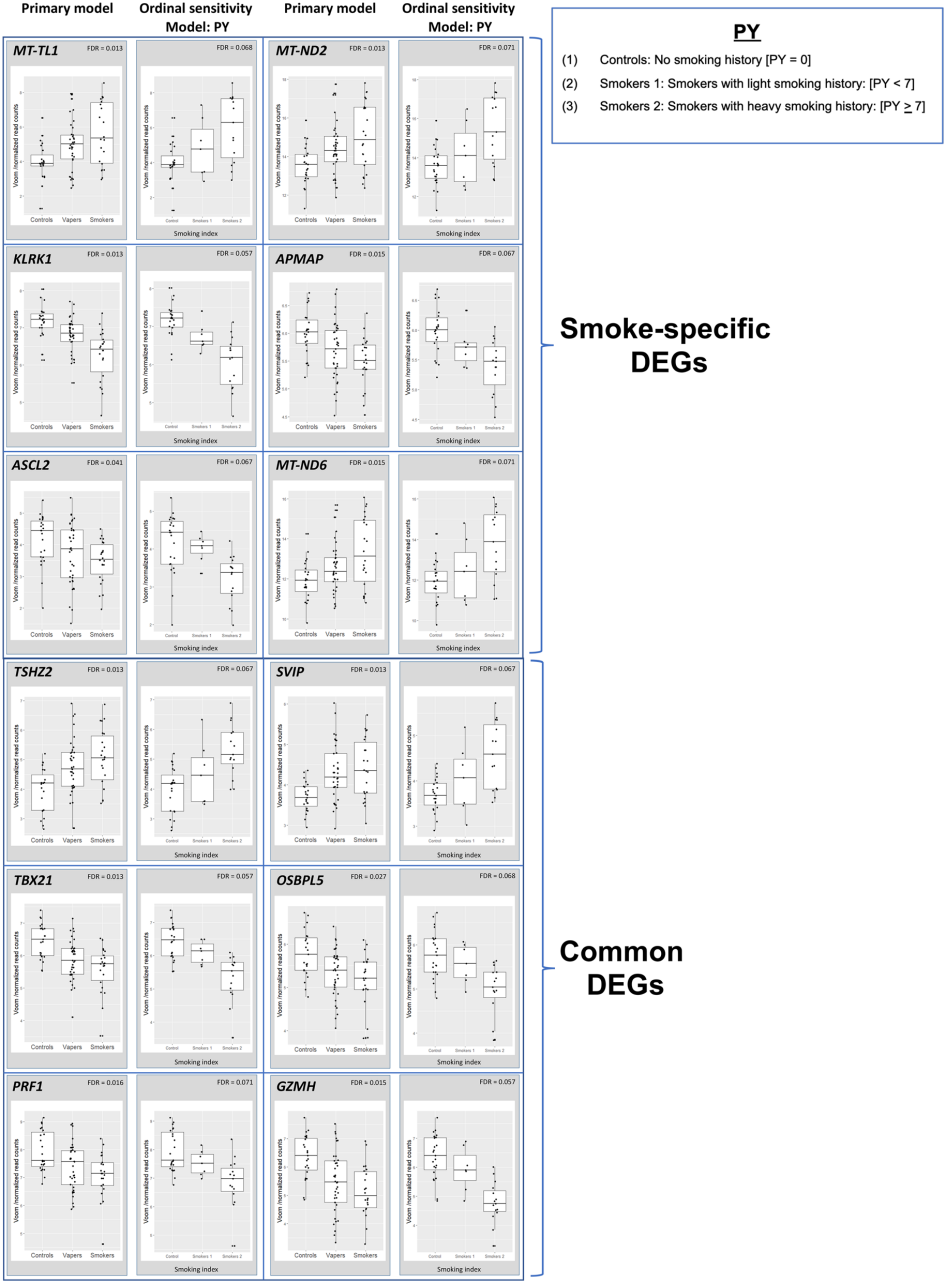
Visualization of the results of primary and ordinal sensitivity analyses—Smoking. Gene expression results for six smoke-specific DEGs (upper panel) and six common DEGs (lower panel), as determined by primary and PY sensitivity analyses, are shown. Concordant statistically significant differential expression results for target genes in primary and PY sensitivity analyses indicates that smoking dose (*i.e.*, intensity and duration of smoking) is significantly associated with gene dysregulation in smokers. In the PY sensitivity model, smokers were divided in two categories, including Light smokers [Smokers 1: PY < 7], and Heavy smokers [Smokers 2: PY ≥ 7] in comparison to Controls [No smoking history: PY = 0] (*see*, also legend for Fig. 2).

Furthermore, we performed PY sensitivity analysis to assess the relationship between DEGs in smokers and smoking dose. Of the 683 DEGs in smokers identified by primary analysis, 218 (31.9%) showed concordant statistically significant differential expression results in PY sensitivity analysis (Supplementary Table S2). One hundred and ninety-four out of 616 (31.5%) smoke-specific DEGs under the primary model, were consistently differentially expressed in smokers when tested by PY sensitivity analysis. Also, 24 out of the 67 (35.8%) common DEGs under the primary model, showed consistent differential expression results under PY sensitivity model (*see*, “Discussion”) (Supplementary Table S2). Figure 3 displays the concordance of gene expression results for six smoke-specific DEGs (upper panel) and six common DEGs (lower panel) in smokers *vs*. controls, as determined by primary and PY sensitivity analyses.

### Molecular pathway and functional network analyses

We used the Ingenuity Pathway Analysis (IPA) to obtain detailed information from the gene lists generated by RNA-seq in vapers and smokers as compared to controls. Of the 92 aberrantly expressed transcripts in vapers, 81 (88.0%) mapped to known IDs, while 523 out of the 683 dysregulated transcripts in smokers (76.6%) had an assigned ID. The ‘Comparison Analysis tool’ in IPA was utilized to identify biological trends or functional similarities and differences in DEGs between vapers and smokers.

**Figure 4:**
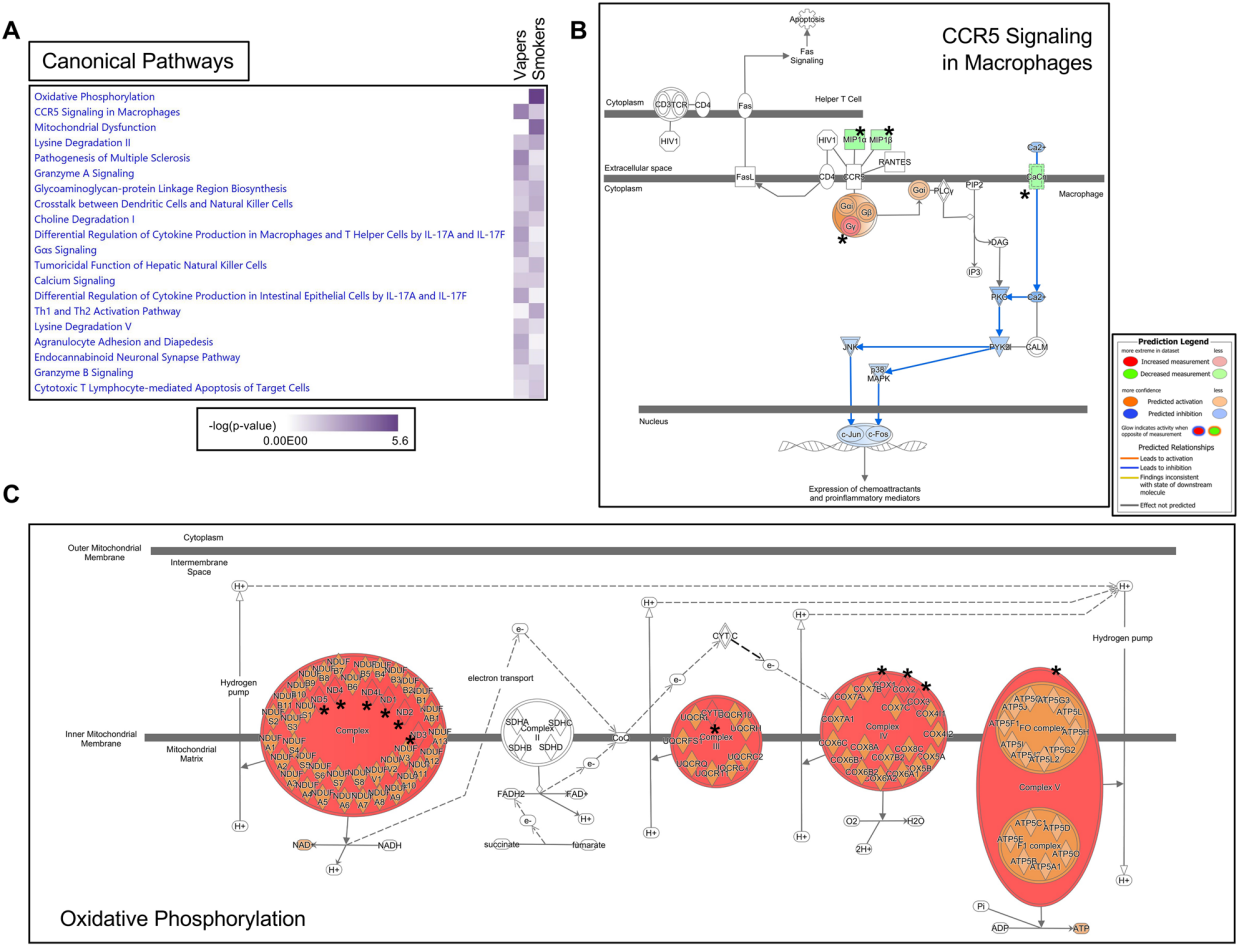
Canonical pathway analysis of differentially expressed genes in vapers and smokers by IPA. **(A)** Comparison Analysis was used to identify trends or similarities and differences across the datasets. The heatmap shows the top twenty canonical pathways impacted in vapers and smokers (based on *P*-value), allowing a direct comparison between the two groups. **(B)** The ‘*CCR5 signaling in macrophages*’ pathway was the top dysregulated pathway in vapers (*P* = 2.15E−04). Affected molecules include CACNA2D2, CCL3/MIP1α, CCL4/MIP1β, and GNG11. **(C)** The ‘*Oxidative phosphorylation*’ pathway was the top disrupted pathway in smokers (*P* = 2.80E−06). Affected molecules include ND1, ND2, ND3, ND4, ND4L, and ND5 (Complex I), Cyt b (Complex III), CO1, CO2, and CO3 (Complex IV), and ATP6 (Complex V). In both cases, Molecule Activity Predictor (MAP) analysis was used to predict how up-regulated and down-regulated genes in the datasets (red and green nodes, respectively) can affect the activity of other molecules on the pathway. For clarity, the affected genes are indicated by asterisks in the two pathways. Orange nodes, prediction of activation; blue nodes, prediction of inhibition.

As shown in Fig. 4A, the ‘*C–C Motif Chemokine Receptor 5 (CCR5) signaling of macrophages*’ was the most affected pathway in vapers, suggesting an interplay between vaping and the inflammatory response (*P* = 2.15E-04). CCR5 is a 41-kDa cell surface G protein-coupled receptor expressed in lymphoid organs, such as thymus and spleen, as well as in peripheral blood leukocytes, specifically T cells, and macrophages^25,26^. Upon interaction with multiple ligands, including the cognate ligands CCL3/MIP1α and CCL4/ MIP1β (transcriptionally down regulated in vapers in our dataset), CCR5 regulates leukocyte chemotaxis in inflammation^25,26^. Based on IPA prediction analysis, CCR5 signaling activity is reduced in vapers (Fig. 4B). In smokers, the ‘*oxidative phosphorylation*’ (OXPHOS) metabolic pathway was greatly dysregulated, affecting 11 of the 13 mitochondrial protein-coding genes (84.6%) (Fig. 4A). These genes encode protein subunits of the enzyme complexes of the oxidative phosphorylation system, which enables mitochondria to act as the powerhouses of our cells^24^. The affected genes express six subunits of the respiratory chain complex I (ND1, ND2, ND3, ND4, ND4L, and ND5), one subunit of complex III (Cyt b), three subunits of complex IV (CO1, CO2, CO3), and one subunit of complex V (ATP6) (*P* = 2.80E−06) (Fig. 4C). The observed increase in MT-gene expression in both smokers and vapers suggests induction of mitochondrial dysfunction and damage in these groups. This may represent a feedback response mechanism in smokers and vapers whereby increased production of proteins involved in the respiratory chain counteracts mitochondrial functional failure (*see*, below).

**Figure 5:**
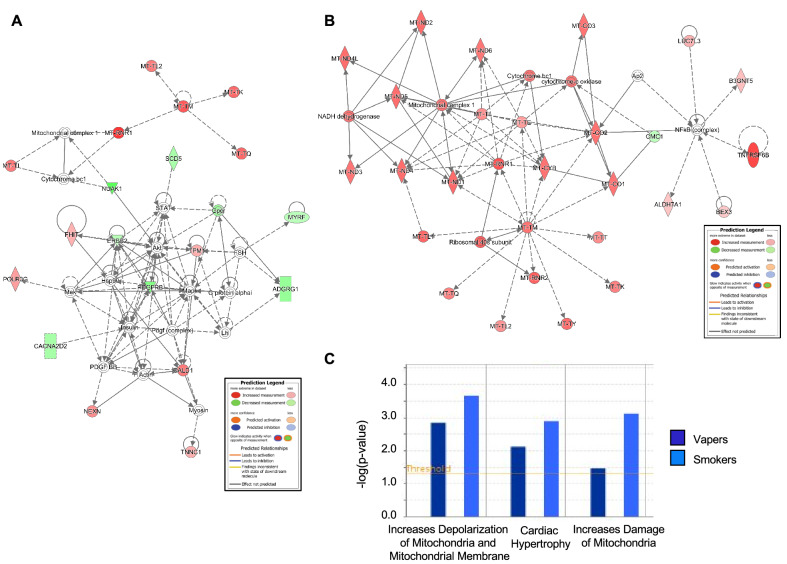
Gene networks and toxicity functions analysis of differentially expressed genes in vapers and smokers by IPA. The top functional networks impacted in **(A)** vapers and **(B)** smokers show high enrichment of mitochondrial genes. Red and green nodes represent up-regulated and down-regulated DEGs, respectively. White nodes show molecules that are not included in the datasets but interact with other components of the network. Solid line, direct interaction; dashed line, indirect interaction. **(C)** The IPA-Tox analysis tool was used to catalogue sets of molecules in the list of DEGs that were known to be involved in a particular type of toxicity or phenotype. Major toxic effects associated with DEGs in vapers (dark blue) and smokers (light blue) include increased depolarization of the mitochondrial membrane and damage of the mitochondria.

Consistent with the high representation of mitochondrial DEGs in both vapers and smokers (Fig. 1B and Supplementary Tables S1 and S2), the top affected functional networks in both groups (Fig. 5A,B) are highly enriched in mitochondrial genes and significantly associated with mitochondrial DNA-related disorders (Table 1). Based on IPA toxicity analysis, both vaping and smoking are linked to ‘*increased depolarization and damage of the mitochondria*’, likely through dysregulation of nuclear genes/proteins involved in maintaining physiological membrane potential (CACNA2D2 and GZMH in vapers, *P* = 1.42E−03; CACNA2D2, FASLG, GZMB, and GZMH in smokers, *P* = 2.16E−04) (Fig. 5C). The transmembrane potential of the mitochondria (∆Ψ_m_) is generated by proton pumps (Complexes I-IV) and represents an essential component in the process of energy storage (through ATP synthesis) during oxidative phosphorylation. Besides its importance in energy production, ΔΨ_m_ also determines the viability of mitochondria, thus allowing elimination of dysfunctional mitochondria by the cells. ΔΨ_m_ is also a driving force for the transport of charged compounds, some of which are essential for mitochondrial viability. Therefore, maintaining normal ∆Ψ_m_ values is crucial for cell homeostasis^27^.

**Table 1:** Top 10 diseases and functions associated with the top disrupted networks in vapers and smokers, respectively, as illustrated in Fig. 5A,B.

	Categories	Diseases or functions annotation	*P*-value	Molecules	# Molecules
Vapers	Hereditary disorder, metabolic disease, neurological disease, organismal injury and abnormalities, psychological disorders, skeletal and muscular disorders	MELAS syndrome	4.71E-13	MT-TI, MT-TK, MT-TL2, MT-TM, MT-TQ	5
	Metabolic disease, organismal injury and abnormalities	Mitochondrial DNA-related disorder	5.05E-12	Mitochondrial complex 1, MT-RNR1, MT-TI, MT-TK, MT-TL2, MT-TM, MT-TQ	7
	Gene expression	Elongation of mRNA	6.67E-12	MT-TI, MT-TK, MT-TL2, MT-TM, MT-TQ	5
	Metabolic disease, neurological disease, organismal injury and abnormalities, skeletal and muscular disorders	Mitochondrial cytopathy	6.43E-09	Mitochondrial complex 1, MT-TI, MT-TK, MT-TL2, MT-TM, MT-TQ	6
	Hereditary disorder, organismal injury and abnormalities, skeletal and muscular disorders	Hereditary myopathy	9.99E-09	CALD1, MT-RNR1, MT-TI, MT-TK, MT-TL2, MT-TM, MT-TQ, NEXN, TNNC1, TPM1	10
	Protein synthesis	Elongation of protein	2.96E-08	Insulin, MT-TI, MT-TK, MT-TL2, MT-TM, MT-TQ	6
	Metabolic disease, organismal injury and abnormalities	Mitochondrial disorder	4.62E-08	Cytochrome bc1, Mitochondrial complex 1, MT-RNR1, MT-TI, MT-TK, MT-TL2, MT-TM, MT-TQ	8
	Gene expression, protein synthesis	Translation of mRNA	7.27E-08	MT-RNR1, MT-TI, MT-TK, MT-TL2, MT-TM, MT-TQ	6
	Cardiovascular disease, hereditary disorder, organismal injury and abnormalities, skeletal and muscular disorders	Familial cardiomyopathy	6.76E-07	MT-RNR1, MT-TI, NEXN, TNNC1, TPM1	5
	Cardiovascular disease, organismal injury and abnormalities, skeletal and muscular disorders	Nonischemic cardiomyopathy	8.84E-07	MT-RNR1, MT-TI, NEXN, TNNC1, TPM1	5
Smokers	Metabolic disease, organismal injury and abnormalities	Mitochondrial DNA-related disorder	5.52E-50	Mitochondrial complex 1, MT-CO1, MT-CO2, MT-CO3, MT-CYB, MT-ND1, MT-ND2, MT-ND3, MT-ND4, MT-ND4L, MT-ND5, MT-ND6, MT-RNR1, MT-TE, MT-TI, MT-TK, MT-TL1, MT-TL2, MT-TM, MT-TQ, MT-TT, MT-TY	22
	Metabolic disease, neurological disease, organismal injury and abnormalities, skeletal and muscular disorders	Mitochondrial leukoencephalopathy	4.13E-47	MT-CO1, MT-CO2, MT-CO3, MT-CYB, MT-ND1, MT-ND2, MT-ND3, MT-ND4, MT-ND4L, MT-ND5, MT-ND6, MT-TE, MT-TI, MT-TK, MT-TL1, MT-TL2, MT-TM, MT-TQ, MT-TT, MT-TY	20
	Metabolic disease, neurological disease, organismal injury and abnormalities, skeletal and muscular disorders	Mitochondrial cytopathy	2.13E-43	Mitochondrial complex 1, MT-CO1, MT-CO2, MT-CO3, MT-CYB, MT-ND1, MT-ND2, MT-ND3, MT-ND4, MT-ND4L, MT-ND5, MT-ND6, MT-TE, MT-TI, MT-TK, MT-TL1, MT-TL2, MT-TM, MT-TQ, MT-TT, MT-TY	21
	Hereditary disorder, metabolic disease, neurological disease, organismal injury and abnormalities, psychological disorders, skeletal and muscular disorders	MELAS syndrome	1.3E-39	MT-CO1, MT-ND1, MT-ND4, MT-ND5, MT-ND6, MT-TE, MT-TI, MT-TK, MT-TL1, MT-TL2, MT-TM, MT-TQ, MT-TT, MT-TY	14
	Neurological disease, organismal injury and abnormalities	Leukoencephalopathy	1.31E-38	ALDH7A1, MT-CO1, MT-CO2, MT-CO3, MT-CYB, MT-ND1, MT-ND2, MT-ND3, MT-ND4, MT-ND4L, MT-ND5, MT-ND6, MT-TE, MT-TI, MT-TK, MT-TL1, MT-TL2, MT-TM, MT-TQ, MT-TT, MT-TY	21
	Metabolic disease, organismal injury and abnormalities	Mitochondrial disorder	9.42E-36	Cytochrome bc1, Mitochondrial complex 1, MT-CO1, MT-CO2, MT-CO3, MT-CYB, MT-ND1, MT-ND2, MT-ND3, MT-ND4, MT-ND4L, MT-ND5, MT-ND6, MT-RNR1, MT-TE, MT-TI, MT-TK, MT-TL1, MT-TL2, MT-TM, MT-TQ, MT-TT, MT-TY	23
	Developmental disorder, hereditary disorder, metabolic disease, neurological disease, ophthalmic disease, organismal injury and abnormalities, skeletal and muscular disorders	Leber optic atrophy	5.73E-30	Mitochondrial complex 1, MT-CO1, MT-CO3, MT-CYB, MT-ND1, MT-ND2, MT-ND3, MT-ND4, MT-ND4L, MT-ND5, MT-ND6, MT-TL1	12
	Hereditary disorder, metabolic disease, neurological disease, organismal injury and abnormalities, skeletal and muscular disorders	Leigh syndrome	1.31E-28	MT-CO1, MT-CO2, MT-CO3, MT-CYB, MT-ND1, MT-ND2, MT-ND3, MT-ND4, MT-ND4L, MT-ND5, MT-ND6, MT-TK, MT-TL1	13
	Developmental disorder, hereditary disorder, metabolic disease, organismal injury and abnormalities	Mitochondrial respiratory chain deficiency	5.23E-24	MT-CO1, MT-CO2, MT-CO3, MT-CYB, MT-ND1, MT-ND2, MT-ND3, MT-ND4, MT-ND5, MT-ND6, MT-TE, MT-TL1, MT-TY	13
	Hereditary disorder, organismal injury and abnormalities, skeletal and muscular disorders	Hereditary myopathy	1.21E-21	MT-CO1, MT-CO2, MT-CO3, MT-CYB, MT-ND1, MT-ND2, MT-ND3, MT-ND4, MT-ND4L, MT-ND5, MT-ND6, MT-RNR1, MT-TE, MT-TI, MT-TK, MT-TL1, MT-TL2, MT-TM, MT-TQ, MT-TT, MT-TY	21

**Figure 6:**
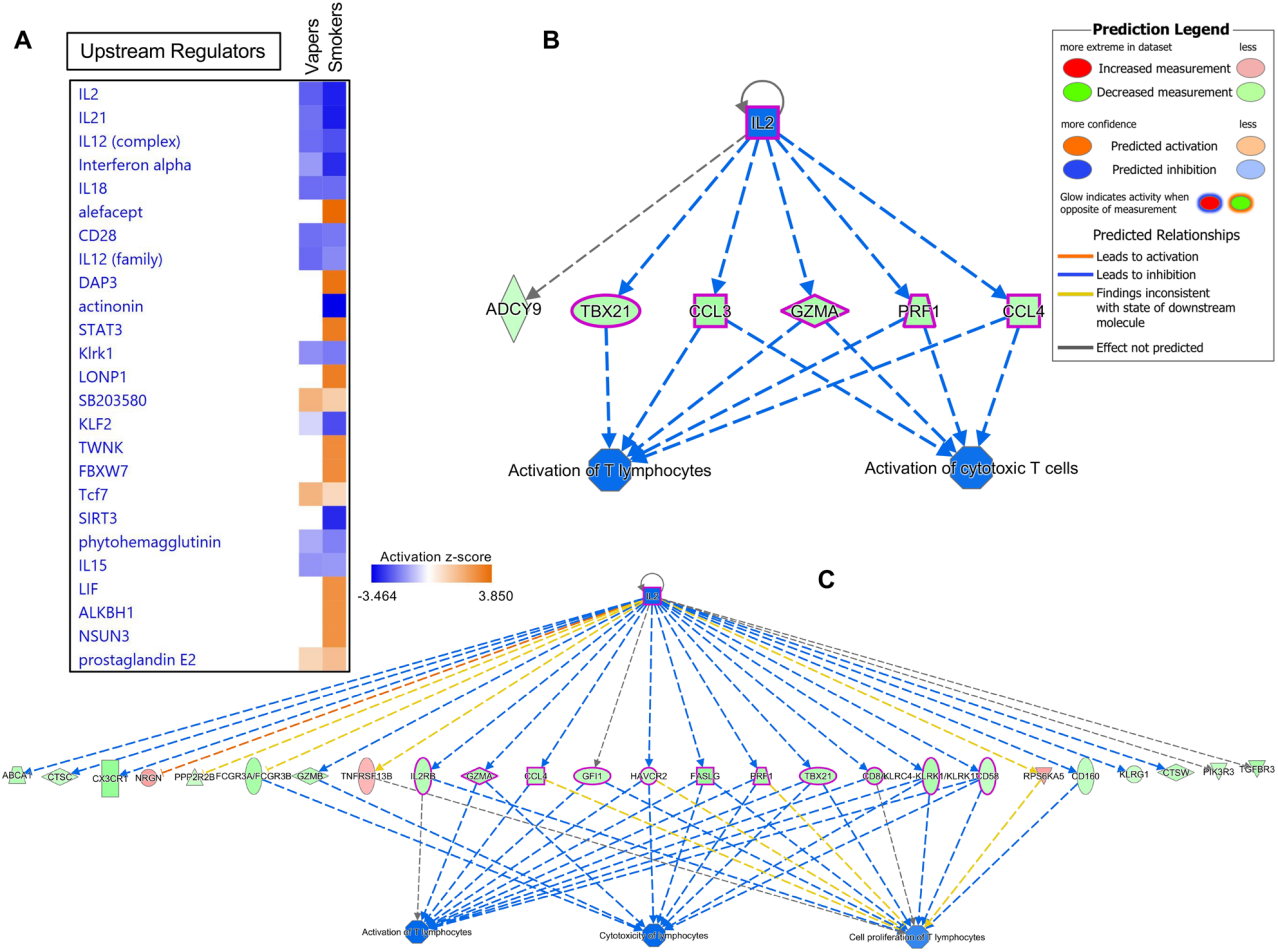
Upstream Regulator Analysis of differentially expressed genes in vapers and smokers. The IPA Upstream Regulator Analysis was used to identify upstream regulators that are likely to account for the aberrant expression of genes identified in vapers and smokers. **(A)** The Upstream Regulator Heat Map for the top 25 upstream regulators is shown. Orange squares indicate predicted increase in regulator’s activity, whereas blue squares indicate predicted decrease in activity. Of interest, the most significant upstream regulators identified in both vapers and smokers are members of a large class of proteins known as cytokines (IL2, IL21, IL12, IFNα, IL18, etc.), which play a crucial role in innate immunity and inflammation. Based on the activation *z*-score, all cytokines in the list are predicted to be inhibited in both vapers and smokers, although the number of affected downstream molecules differs between the two groups. **(B)** Regulatory network of IL2, its targeted genes and downstream biological effects in vapers. In vapers, inhibition of IL2 is likely to lead to downregulation (indicated by blue lines) of genes (shown by green color), which in turn may lead to impaired immune response (*i.e.*, lack of activation of T lymphocytes). **(C)** Regulatory network of IL2, its targeted genes and downstream biological effects in smokers. Likewise, in smokers, inhibition of IL2 is likely to disrupt normal immune functions, though the number of genes modulated by IL2 is much higher in smokers than vapers (25 *vs*. 6). For more indicators, please refer to the Prediction Legend.

Comparison Analysis in IPA was further employed to identify upstream regulators, including transcription factors and chemicals, that were predicted to be activated or inhibited based on the expression status of DEGs in the two datasets. Figure 6A shows the top 25 upstream regulators that are likely to modulate the expression of genes impacted in vapers and smokers, respectively. While there are remarkably similar trends in modulation of DEGs in vapers and smokers, the number of downstream effectors varies between the two groups (Fig. 6B,C). Common master molecules include members of a large class of proteins known as cytokines (IL-2, IL-21, IL-12, IFN-α, IL-18, etc.), which play a crucial role in innate immunity and inflammation (Fig. 6A)^28–30^. Based on the activation *z*-score (Fig. 6A), all cytokines in the list are predicted to be inhibited in both vapers and smokers, although the number of affected downstream molecules differs between the two groups. For instance, interleukin 2 (IL-2), the top master regulator, is likely to influence the expression of 6 downstream targets in vapers and 25 targets in smokers, most likely leading to ‘*inhibition of T-lymphocytes response*’ (Fig. 6B,C).

Altogether, our IPA analysis shows that vapers, similarly to smokers, exhibit disruption of key functional pathways and gene networks in peripheral blood leukocytes. Notably, mitochondrial dysfunction and impaired innate immunity (inflammatory response) are highly associated with the DEGs detected in both vapers and smokers, although the extent of effects differs between the two groups. Supplementary Figure S1 summarizes the results of Top Diseases and BioFunctions analysis of DEGs in vapers and smokers. Table 1 lists major diseases and/or function annotations associated with the top disrupted gene networks in vapers and smokers, respectively.

### Validation of RNA-seq data by RT-qPCR

To independently validate the RNA-seq expression results, we randomly selected several of the identified up- and down-regulated genes in vapers and smokers, and examined their transcription levels by RT-qPCR. Supplementary Figure S2 shows the correlation results for expression of the tested genes, as determined by RT-qPCR *vs.* RNA-seq. In all cases, the median normalized expression levels of the target genes determined by RT-qPCR were directly correlated to the normalized read counts in RNA-seq. Thus, we have validated the RNA-seq expression data by RT-qPCR analysis using triplicate samples from our study population. We stress that the Illumina sequencing data are proven to be highly replicable, with few systematic differences among technical replicates^31,32^. Therefore, for most applications, it suffices to sequence each mRNA sample only once^31^, considering the limiting source materials (tissue/cells), especially in population-based studies^32^. It is well-established that adding more technical replicates gives diminishing return on accuracy and statistical power to detect DEGs^32,33^. Conversely, adding biological replicates (*i.e.*, more samples) and increasing the sequencing depth (up to a certain level) generate more informational reads, thereby significantly improving the sensitivity and statistical power to detect DEGs^32^.

### Verification of vaping/smoking status

To verify smoking/vaping status, we measured the concentrations of cotinine, a major metabolite of nicotine^5^, in blood plasma of our study subjects. Overall, cotinine levels in both vapers and smokers were significantly higher than those in controls (vapers: 115.0 ± 9.1 ng/ml, smokers: 121.0 ± 11.2 ng/ml, controls: 2.5 ± 0.1 ng/ml, *P* = 3.56E-9 and *P* = 8.91E-9, respectively). The levels of cotinine in vapers and smokers were not significantly different from one another (*P* = 0.82). To further validate the e-cig/cigarette use and frequency data obtained during in-person interviews, we sought correlations between subjects’ cotinine levels and the self-reported vaping/smoking indices (cum e-liq/PY). We observed a positive and statistically significant correlation between the detected levels of cotinine and self-reported cumulative e-liquid consumption levels in the study subjects (*r* = 0.78, *P* = 1.61E−13). Likewise, a direct and statistically significant correlation was observed between the measured levels of cotinine and self-reported pack years in the study subjects (*r* = 0.81, *P* = 1.03E−11). Altogether, our objective measurement of plasma cotinine in this study population is highly consistent with their self-reported vaping/smoking indices, as recorded during personal interviews.

## Discussion

In the present study, we have compared the biological consequences of e-cig use and cigarette smoking by constructing and analyzing the whole transcriptome in leukocytes of healthy adult vapers (with and without a history of smoking), exclusive cigarette smokers, and control nonsmokers non-vapers. Transcriptome analysis in peripheral blood leukocytes has been widely used to study the regulation of genes in a variety of diseases, including cardiovascular disease, immune-related (inflammatory) disease, respiratory disease, and cancer^1,5,12,13^. Specifically, gene expression analysis in leukocytes has been extensively exploited for investigating the effects of exposure to inhaled chemicals, such as tobacco smoke^1,5,34^. Through systemic circulation, blood cells interact with key organs, such as the lungs (in capillaries), liver (in sinusoids), and kidneys (in glomerus capillary plexus)^35^, all of which are major targets for tobacco-related diseases and inflammatory conditions and disorders^13,36^.

Our limmaVoom analysis of RNA-seq data showed significant dysregulation of functionally important genes and molecular pathways in both vapers and smokers as compared to controls (adjusted for sex and age). Bioinformatics analysis and computational modeling, combining primary and sensitivity analyses, revealed that e-cig use, but not past smoking, is significantly associated with gene dysregulation in vapers. A substantial portion of the dysregulated genes in vapers (81.5%) and nearly one-third of all DEGs in smokers were dose-dependently associated with vaping and smoking, respectively. Specifically, there were direct relationships between differential expression of genes in vapers and smokers and cumulative exposure to e-cigs and tobacco cigarettes, respectively. We note that the higher number of vapers than smokers (37 *vs.* 22) together with the more balanced distribution of sex and age within the vaping group may have contributed to the stronger associations found between vapers’ DEGs and e-cig exposure index (cum e-liq) than smokers’ DEGs and the intensity and duration of smoking (PY). In confirmation, the average power to detect DEGs in vapers when controlling FDR^21^ was 65% at an adjusted *P*-value of 0.0476, whereas in smokers, the average power to detect DEGs while controlling FDR^21^ was 54% at an adjusted *P*-value of 0.0499. Furthermore, because cigarette smoke contains several thousand chemicals, many more than those present in e-cig vapor (and mostly at substantially higher levels)^1,2,8^, establishing a dose–response relationship for smokers may prove more complicated than for vapers. These scenarios should be investigated in future studies with larger sample size, when specimens from well-characterized vapers and smokers, with varying tobacco product use frequency and patterns, will be analyzed.

Comparative analysis of the gene networks and canonical pathways impacted in vapers and smokers showed strikingly similar biological outcomes, though the number of affected genes varied considerably between the two groups. Importantly, a significant percentage (12.0%) of DEGs in vapers consisted of mitochondrial genes, including one MT-rRNA and 10 MT-tRNAs (all over-expressed) (Supplementary Table S1), suggesting that vaping interferes with mitochondrial homeostasis. Likewise, 32 of all 37 mitochondrial genes^24^, including 13 protein-coding genes, two MT-rRNAs and 17 MT-tRNAs, were up-regulated in smokers (Supplementary Table S2). Notably, IPA analysis of the dysregulated genes in vapers and smokers confirmed a high enrichment of mitochondrial genes in both groups (Fig. 5A,B). Furthermore, major diseases and/or function annotations associated with the top disrupted gene networks in both vapers and smokers included several mitochondrial disorders that are characterized by structurally, functionally, or numerically abnormal mitochondria (Table 1)^37^. This together with the observed up-regulation of mitochondrial genes in both vapers and smokers is suggestive of occurrence of mitochondrial dysfunction and damage in both groups.

Mass spectrometry analysis of a wide range of e-cig liquids and aerosols has demonstrated the presence of aldehydes, free radicals, and heavy metals, which are known mitochondrial toxicants^1,2,5,7,8,38^. Unlike cigarette smoke whose deleterious effects on mitochondria have been well-established^38–41^, data on vaping-associated mitochondrial dysfunction remain scarce, and mostly limited to cell lines and animal models^42,43^. Recent studies in mouse and human cells have shown that exposure to e-cig aerosols or liquids induces cytotoxicity by impairing mitochondrial membrane potential (∆Ψ_m_) and generating reactive-oxygen species (ROS). These effects are similar to those caused by chronic exposure to cigarette smoke^42–44^. Consistent with the findings of those reports^42–44^, our data support that vaping and smoking may increase depolarization of mitochondria and mitochondrial membrane (Fig. 5C), which can subsequently cause ATP reduction and eventually lead to cell injury or death^27,39^.

Another salient finding of our study is that vaping and smoking impact innate immunity and inflammatory response, although to varying degrees. As shown in Fig. 6A, aberrant expression of the immune response genes in vapers and smokers is likely to be modulated by several upstream cytokines, including members of the interleukin (IL) and interferon (IFN) family, that play a crucial role in innate immunity and inflammation (Fig. 6A)^28–30,45^. One of such cytokines is interleukin 2 (IL-2), which is produced by activated CD4 + and CD8 + T lymphocytes and is important for the proliferation of T and B cells (Fig. 6B,C). Among its multiple, often competing, roles in immunity, IL-2 functions as an anti-inflammatory cytokine by preventing the uncontrolled expansion of immune response (through production of regulatory T cells) and suppressing overall inflammation^29,30^. Of note, patients lacking IL-2 expression exhibit a defective immune response^30^. Furthermore, in mice, targeted disruption of a gene similar to IL-2 leads to ulcerative colitis-like disease. These observations support an essential role for IL-2 in eliciting the immune response to antigenic stimuli^30^. Thus, it is conceivable that any suppression of the immune system and inflammatory response triggered by inhibition of IL2 and/or other cytokines (Fig. 6B,C) may lead to increased susceptibility to infections and/or increased severity of infections in vapers, much like smokers. Consistent with our findings, a recent study has shown that both e-cig use and cigarette smoking were associated with decreased expression of several immune and inflammatory-response genes in nasal epithelial cells of vapers and smokers, causing disruption of normal immune functions^46^. Furthermore, e-cig use has been linked with increased risk of suppressed host-defense functions in response to bacterial infection^47^ or following infection with live-attenuated influenza virus^48^. The suppressed immune response associated with vaping, manifested as altered immune-gene expression, cytokine and chemokine release, and antibody production^47,48^. Follow-up mechanistic studies are needed to establish the chain of events leading to the dysregulation of functional pathways and gene networks in vapers and smokers, as identified in the present study.

Growing evidence is emerging on the central role of mitochondria as signaling organelles, which govern fundamentals of immunity and inflammatory response^49–52^. Mitochondria can regulate innate and adaptive immunity through distinct mechanisms^49^. One way is through the release of damage-associated molecular patterns (DAMPs), which include mitochondrial DNA (mtDNA), ATP, cardiolipin and formyl peptides^53^. Owing to the bacterial ancestry of mitochondria, mtDAMPs are recognized by the same set of innate immune receptors (*e.g.*, Toll-like receptor 9), which detect bacterial infections and trigger an inflammatory response (*i.e.*, chemotaxis of innate immune cells and cytokine production)^49,54^. Notably, a positive association has been found between leukocyte mtDNA content and risk of coronary heart disease, which is strongly associated with smoking^55^. Leukocyte mtDNA copy number content in cancer patients has also been shown to associate with levels of leukocyte 8-hydroxy-2′-deoxyguanosine (8-OHdG), a biomarker of oxidative DNA damage^56^. Mitochondrial metabolic pathways, such as tricarboxylic acid cycle, OXPHOS, and fatty acid oxidation can also have a major impact on immune cell activity, and are important for macrophage polarization and T cell differentiation^49,51^. Activation of T cells relies on functional OXPHOS for their bioenergetic requirements (consistent with data in Fig. 4C)^51^. Other components of the mitochondrial machinery that play crucial roles in immunity and inflammation include amino acid metabolism, antioxidant systems, mitochondrial dynamics, mitophagy, and mtROS production^49^. Persistent mitochondrial dysfunction can cause chronic inflammation, which in turn may lead to several chronic inflammatory disorders, including cardiovascular, respiratory (COPD), and metabolic diseases, as well as cancer^52,57^.

While the novel findings of the present study have significant implications for public health and regulation of tobacco products, we also acknowledge the limitations of our study, in terms of its representativeness for the general population. Future studies with larger sample size should verify the generalizability of our findings to the broad population of vapers and smokers. These follow-up studies should also investigate the health consequences of vaping combined with other lifestyle habits, including co-use with recreational drugs. Of note, marijuana vaping is on the rise, particularly among youth and young adults^58,59^.

In summary, we have demonstrated preferential targeting of the mitochondrial genes, important for innate immunity and inflammatory response, in peripheral blood leukocytes of vapers and smokers. We have also shown that e-cig use, but not past smoking, is significantly associated with dysregulation of gene transcription in chronic vapers. Together with the observation that most dysregulated genes in vapers (72.8%) are common to those found in smokers, our findings support that gene dysregulation in vapers is likely due to exposure to similar chemical(s) present in both e-cig vapor and cigarette smoke. Although the exact identity of these chemicals remains to be determined, potential candidates may include ROS-inducing chemicals and/or heavy metals^13^. Future studies are warranted to identify the constituents of e-cig vapor that are responsible for the observed dysregulation of genes in vapers, similarly to smokers. Lastly, we have shown accentuated transcriptomic effects in smokers relative to vapers, suggesting that smoking has greater and more pronounced adverse effects than vaping on biological systems. Altogether, the results of this research and future investigations into the health risks or potential benefits of vaping *vs*. smoking should provide scientific evidence to inform the regulation of tobacco products to protect public health.

## Methods

### Ethics declarations

The study was approved by the Health Sciences Institutional Review Board (IRB) of the University of Southern California (Protocol No: HS-16-00175). Written informed consent was received from participants prior to inclusion in the study. All research was performed in accordance with the approved IRB protocol and relevant guidelines & regulations, including the Declaration of Helsinki.

### Study population

Eligible candidates for the study included healthy adults—both males and females of diverse ages, races, and ethnicities—who could read and write in English and understand and give informed consent. The catchment area for this study was the Greater Los Angeles Area. The study population consisted of 82 subjects divided into three groups, including Group 1: current vapers (*n* = 37), Group 2: current smokers (*n* = 22); and Group 3: control nonsmokers non-vapers (*n* = 23). Detailed characteristics of the study population are listed in Table 2. Dual users of e-cigs and combustible cigarettes or poly users of e-cigs, cigarettes, or other tobacco products were excluded from the study. Criteria for classification of the study subjects, as vapers, cigarette smokers, or controls, were as follows: vapers were those who reported current use of e-cigs for at least three times a week for a minimum of six months, and no use of conventional cigarettes or any other tobacco products in the past six months. Smokers were those who reported current smoking of tobacco cigarettes at least three times per week for a minimum of one year, and no use of any other tobacco products, including e-cigs, in the past six months. Controls were those who reported no use of any tobacco product (e-cigs or combustible) more than five times in their life, with no use in the past six months; controls reported smoking no or fewer than 100 cigarettes or having no or less than five vaping sessions in their lifetime. We note that unlike combustible cigarettes that have been in the market for many years, e-cigs are a relatively new tobacco product. Therefore, we set the minimum use criteria for vapers and smokers to six months and one year, respectively, to allow enrollment of sufficient number of subjects into this study^15^.

**Table 2:** Characteristics of the study population.

	Vapers (*n* = 37)	Smokers (*n* = 22)	Controls (*n* = 23)
Age*	28.0 ± 1.5 (Range: 21–56)	36.5 ± 2.9 (Range: 24–66)	24.0 ± 1.9 (Range: 22–58)
**Gender**^†^			
Male	30 (81.1%)	17(77.3%)	13 (56.5%)
Female	7 (18.9%)	5(22.7%)	10 (43.5%)
**Race**^†^			
White	14 (37.9%)	5 (22.7%)	2 (8.7%)
Hispanic	10(27.0%)	1 (4.5%)	5 (21.8%)
African American	5(13.5%)	8(36.4%)	2(8.7%)
Asian	6 (16.2%)	4 (18.2%)	11 (47.8%)
Other^‡^	2 (5.4%)	4(18.2%)	3 (13.0%)
BMI*^,§^	27.2 ± 1.1	27.6 ± 1.0	23.9 ± 1.4
Pack year*^,¶^	5.0 ± 2.2	10.3 ± 2.3	NA
Cumulative e-liquid (ml)*^,#^	5096.0 ± 3446.5	NA	NA
E-cig device type^†,||^		NA	NA
1st Generation	3 (8.1%)		
2nd Generation	2 (5.4%)		
3rd Generation	23 (62.2%)		
4th Generation	0 (0%)		
Multiple	1st and 3rd: 1 (2.7%); 2nd and 3rd: 7 (18.9%); 1st, 2nd, and 3rd: 1 (2.7%)		
Plasma cotinine (ng/ml)*	115.0 ± 9.1	121.0 ± 11.2	2.5 ± 0.1
Years smoked*	8.0 ± 1.6	21.0 ± 2.7	NA
Years vaped*	3.0 ± 0.3	NA	NA
Elapsed time (years) since last cigarette smoked*	2.0 ± 0.7	NA	NA

*NA* not applicable.

*Results are expressed as Median ± SE.

^†^Numbers and percentages (inside brackets) are indicated.

^‡^Other = Multiracial or Native American.

^§^BMI: Body Mass Index [Weight _(kg)_ ÷ Height^2^_(m)_].

^¶^Pack Year is calculated by multiplying the number of packs of cigarettes a person smoked per day by the number of years he/she smoked.

^#^Cumulative e-liquid is calculated as the total volume of e-liquid (in milliliter) vaped by a person during his/her lifetime.

^||^Device types are divided into 1st Generation: Cig-a-Like, disposable; 2nd Generation: Vape Pen, mid-size (laser pointer) with pre-filled or re-fillable cartridges; 3rd Generation: Mod or Tank, large size; 4th Generation: Pod, Pod Mod, or Pod-type, small-size, USB-shaped or other sleek designs, pre-filled or re-fillable (JUUL, JUUL-*alike*); and Multiple: a combination of different generation devices.

### Subject recruitment and enrollment

The study was advertised in online forums, including Craigslist, Reddit, and myUSC (http://my.usc.edu), and on social media (Twitter, Instagram, and Facebook). Also, flyers and leaflets were used to advertise the study in local colleges, universities, and vape shops. Furthermore, an online survey was developed, validated, and subsequently employed to solicit and query potential participants (http://geteo.usc.edu). Individuals who appeared to have met the study criteria were contacted by phone to complete a screening questionnaire. Based on the information obtained during the phone screen, those who were deemed potentially eligible, were scheduled for an in-person visit to our laboratory. During the visit, an expanded version of the phone screen was administered to reconfirm eligibility, and informed consent was obtained, afterwards (*see*, below)^15^.

### Personal interview

Upon reconfirmation of eligibility and informed consent, all participants underwent a personal interview to provide detailed information on demographics, socio-economic status, consumption of e-cigs, cigarettes, or other tobacco products, dietary habits, lifestyle, use of recreational or illicit drugs, alcohol, and prescription- or over-the-counter medicine, occupational and residential history, and family history of disease^15^.

### Inclusion and exclusion criteria

Health indicators for exclusion from the study consisted of respiratory diseases (*e.g.*, asthma or chronic obstructive pulmonary disease (COPD)), immune system disorders, diabetes, kidney diseases, body mass index < 18 kg/m^2^ or > 40 kg/m^2^, local or systemic inflammation or infection, or any medical disorder/medication that could affect subject’s safety or study results. Any unstable or significant medical condition in the past 12 months, including but not limited to symptomatic heart conditions, stroke, severe angina, and hypertension was ground for exclusion. Being pregnant or having a baby in the past 12 months was also exclusionary. Other exclusion criteria included uncontrolled mental illness or substance abuse or inpatient treatment for those conditions in the past 12 months, use of recreational or illicit drugs (*e.g.*, marijuana, heroin, etc.) in the past six months, and use of any medication known to induce/inhibit CYP450 2A6 enzyme. Physical examination and health assessment of all participants were performed by highly trained staff during the personal visits and interviews^15^.

### Sampling and processing of peripheral blood

Peripheral blood (30 ml) was drawn from the study subjects by venipuncture. Plasma was collected by centrifugation, and subsequently leukocytes and erythrocytes were separated using Leucosep tubes according to the manufacturer’s instructions (Greiner Bio-One Inc., Monroe, NC). The collected plasma, and leukocyte and erythrocyte fractions were aliquoted into multiple microtubes (Eppendorf, Inc., San Diego, CA), snap frozen, and preserved at – 80 °C until further analysis. An aliquot of leukocytes from each subject was used for total RNA isolation using the RNeasy Mini Kit (Qiagen, Valencia, CA)^12^.

### RNA-Seq analysis

#### Quality control, library preparation, and sequencing

Total RNA isolated from leukocytes was checked for quality control using the RNA 6000 Nano Chip kit in an Agilent 2100 Bioanalyzer (Agilent Technologies, Santa Clara, CA, USA). Libraries for RNA-seq were prepared from total RNA (300 ng per sample) using the KAPA Stranded mRNA-Seq Kit (Kapa Biosystems, Inc. acquired by Roche). The workflow consisted of mRNA enrichment and fragmentation, first strand cDNA synthesis using random priming, followed by second strand synthesis converting cDNA:RNA hybrid to double-stranded cDNA (ds cDNA), and incorporation of dUTP into the second cDNA strand. cDNA generation was followed by end repair to generate blunt ends, A-tailing, adaptor ligation, and PCR amplification. Different adaptors were used for multiplexing samples in one lane. Sequencing was performed on Illumina Nextseq500 for a single-end read for 75 cycles. Data quality check was done by Illumina SAV. Demultiplexing was performed with Illumina Bcl2fastq2 v2.17 program. To rule out any potential bias, library construction and data acquisition for samples from different groups (vapers, smokers, and controls) were done in the same run, not in different batches, and in a ‘blind’ fashion^15^.

#### Preprocessing of sequencing data, normalization, and variance scaling

RNA-seq data were trimmed, aligned, and quantified using Partek Flow version 8.0.19.1027 (Partek Inc., St. Louis, MO). Raw sequencing reads were trimmed from both ends (Phred QC score ≥ 20, minimum read length = 25 nt). To align trimmed reads to the human reference genome hg38, STAR version 2.6.1d was used with default parameter settings^60^. STAR is a widely used open-source RNA-seq mapper to align empirical or simulated sequence reads to a reference genome with high accuracy and at ultra-fast speed^61^. STAR enables detection of annotated and novel splice junctions, as well as discovery of more complex RNA sequence arrangements, such as chimeric (fusion) and circular RNA. STAR generates output files that can be used for many downstream analyses, such as transcript/gene expression quantification, differential gene expression, novel isoform reconstruction, signal visualization, and so forth^60^. In an elegant study published recently, Donato et al*.*^62^ have benchmarked 17 different aligners (including STAR) in terms of efficiency, accuracy, duplication rate, saturation profile, and running time. They concluded that each aligner excelled in specific area(s), with *“no best aligner among all of the analyzed ones; each tool was the-best in specific conditions”*^62^. Mapped reads were quantified using Partek E/M, Partek’s optimization of the expectation–maximization algorithm for transcript abundance estimation with Gencode 27 annotation^63^. Post-alignment processing, quantification, and downstream differential expression (DE) analysis were performed in R (https://rstudio.com/). To establish quality control, we applied the following criteria: samples were included in the analysis if they had high sequencing depth (> 30 million reads/sample) and high percentage reads aligned (> 50% reads aligned)^15^. All samples passed quality control and met the above criteria. Furthermore, we excluded genes with less than 1 count per million (cpm) in at least 23 samples, where 23 was the sample size of our control group, against which the vaping and smoking groups were contrasted.

Limma-based methods require transformation of count data before entering them into the limma pipeline, a toolkit with statistical methods to perform differential gene expression analysis on microarray- or RNA-seq data^19^. In the present study, we used the limmaVoom with quality weights method^19^ to transform the RNA-seq data and perform variance modeling at the sample and observational levels. This transformation improves detection of differential gene expression by enhancing the capture of transcriptomic variance^19–22^. To adjust for library size variations at the logarithmic scale, we first applied the trimmed mean of M-values (TMM) method^64,65^ to normalize read counts. We used limmaVoom to model the mean–variance relationship of the log2-transformed counts at the individual observation level^19–22^. LimmaVoom with quality weights also improves this procedure by estimating the mean–variance trend at the gene level to account for variations in sample quality^19^. By combining the observational weights with sample weights, this approach accounts for mean–variance relationship in log-transformed counts^19^. Applying this approach, we smoothed the variance due to latent confounding factors in our sample population, thus ensuring an approximately normal distribution of the transformed count data. Feeding the normalized counts and their associated weights into the limma pipeline, we calculated gene-wise log2-fold-changes, with increased statistical power, to estimate the relative RNA expression in each sample^19,20,22^. This allowed us to compute fold-change differences in expression of genes, genome-wide, between different groups, including vapers *vs*. controls and smokers *vs*. controls.

### Differential gene expression analysis: primary model

To detect DEGs in vapers and smokers *vs.* controls, while adjusting for age and sex as covariates, we used the R/Bioconductor limma package^19^ under an empirical Bayes moderated *t*-test^20–22^. We used the limmaVoom with quality weights method to transform the RNA-seq data and account for mean–variance relationship of counts prior to linear modeling in limma^19^. This approach reduces variance due to latent confounding, and ensures an approximately normal distribution of gene expression data^19^. We performed group comparison of DEGs in vapers and smokers relative to controls using linear contrasts defined a priori^19–22^ as follows: tested features were considered differentially expressed if they possessed an absolute fold change (FC) of greater than 1.5 and a false discovery rate (FDR) below 10%.

$$y_{ij} = \alpha_{i} + \beta_{i} Classification_{j} + \delta_{i} Age_{j} + \mu_{i} Sex_{j} + \varepsilon_{ij}$$

We used the above formula for transcript feature *i* and donor *j* and *Classification*_*j*_ (levels: controls, vapers, and smokers), adjusting for subject’s age and sex. After fitting the linear model to RNA-seq data, we applied Empirical Bayes smoothing to the standard errors, borrowing information from all genes^19–22^. The normalized expression data were analyzed in two contrasts relative to control group (*i.e.*, vapers *vs*. controls and smokers *vs*. controls) in such way that regression coefficients represent log-fold changes between comparison groups. We corrected for multiple testing and obtained adjusted *P*-values (FDR) by applying the Benjamini and Hochberg procedure^21^.

### Ordinal sensitivity analysis: sensitivity model

We performed post hoc ordinal sensitivity analysis^23^ to seek the relationship between DEGs in vapers and smokers and exposure indices, including cumulative e-liquid consumption and pack year. Whereas cumulative e-liquid consumption was calculated as the total volume of e-liquid (in milliliter) vaped by a person during his/her lifetime, pack year was estimated by multiplying the number of packs of cigarette a person smoked per day by the number of years he/she smoked^15^. We performed two separate sets of ordinal sensitivity analysis as follows: (I) to assess the persistency of the effects of past smoking on gene expression in vapers ex-smokers, we sought the association between DEGs and pack year; (II) to confirm the consistency and robustness of our analysis, we examined the dependence of DEGs in vapers and smokers on vaping and/or smoking indices, *i.e.*, cumulative e-liquid consumption and pack year.

To tease out the influence of e-cig use in vapers with and without a history of smoking, we stratified vapers into three categories based on pack years (PY) smoked: (Vaper 1) No smoking history [PY = 0; *n* = 7]; (Vaper 2) Light smoking history [PY < 7; *n* = 15]; and (Vaper 3) Heavy smoking history [PY ≥ 7; *n* = 15], with Controls who had no smoking or vaping history. To assess dose-dependent effects of e-cig use in vapers, we also divided vapers in two categories based on cumulative e-liquid (cum e-liq) consumed: (1) Light vapers [cum e-liq < 5000 ml; *n* = 18]; (2) Heavy vapers [cum e-liq ≥ 5000 ml; *n* = 19], with Controls who had no vaping or smoking history. To evaluate dose-dependent effects of cigarette smoking in smokers, we similarly divided smokers in two categories based on PY smoked: (1) Light smokers [PY < 7; *n* = 7]; (2) Heavy smokers [PY ≥ 7; *n* = 15], with Controls who had no smoking history. Testing of the ordinal variables for differential gene expression was performed as described above for primary DE analysis, while adjusting for age and sex. We applied two sensitivity models for vapers, including cum e-liq sensitivity and PY sensitivity models, and one sensitivity model for smokers, including PY sensitivity model. In vapers, we tested whether each DEG identified in our primary analysis remained differentially expressed, at a statistically significant level, in the cum e-liq- and PY sensitivity analyses. Consistent differential expression of the tested DEG in the cum e-liq sensitivity model and failure to remain significantly differentially expressed in the PY sensitivity model indicate that vaping, but not past smoking, contributes to differential expression of the DEG, as detected in the primary analysis. In other words, consistency between the results of our primary analysis and cum e-liq sensitivity analysis, but not PY sensitivity analysis, supports that past smoking in vapers ex-smokers has little to no impact on differential expression of the tested genes. At the same time, it reaffirms that exposure to e-cigs influences differential expression of the tested DEG in vapers, irrespective of past smoking history. Likewise, in smokers, reproducibility of the expression results between our primary analysis and PY sensitivity analysis reassures that exposure to cigarette smoke modulates differential expression of the tested DEG.

### IPA analysis

Functional identification of gene networks, canonical pathways, and upstream regulators was performed using the QIAGEN’s Ingenuity Pathway Analysis (IPA v. 2020; QIAGEN Bioinformatics, Redwood City, CA; www.ingenuity.com). DEG lists from the limma package for vapers and smokers (Supplementary Tables S1 and S2) were uploaded onto IPA for data analysis, as described previously^15^.

### Reverse transcription quantitative PCR (RT-qPCR)

We used a standard RT-qPCR protocol^12^ to validate the expression results of individual genes determined as up-regulated or down-regulated by RNA-seq analysis. Briefly, total RNA (250 ng) from leukocytes was reverse transcribed into cDNA using the iScript™ Reverse Transcription Supermix (iScript RT Supermix) (Bio-Rad laboratories, Inc., Hercules, CA). The synthesized cDNA was diluted 2.5-fold with low-EDTA TE buffer (10 mmol/l Tris–HCl, 0.1 mmol/l EDTA, pH 8.0), of which two microliters were used per reaction in a mastermix containing gene-specific primers and SsoAdvanced Universal SYBR Green Supermix (Bio-Rad laboratories, Inc.). The human actin beta (*ACTB*) gene and the human glyceraldehyde-3-phosphate dehydrogenase (*GAPDH*) gene were used as references. All assays were performed using the CFX96 Touch™ Real-Time PCR detection system (Bio-Rad Laboratories, Inc.). The cycling conditions included a pre-incubation at 95 °C for 2 min, followed by 40 cycles at 95 °C for 5 s, and 58 °C for 30 s^15^. Five randomly selected samples per biological group (*i.e.*, vapers, smokers, and controls) were run in triplicate for a total of 45 reactions for each gene of interest. Relative transcript levels in each sample were calculated using the Bio-Rad CFX Maestro™ software (BioRad Laboratories, Inc.). The primer sets used for RT-qPCR are available upon request.

### Plasma cotinine measurement by ELISA

Plasma cotinine was measured by a solid phase competitive ELISA kit according to the instructions of the manufacturer (Abnova Corp., Walnut, CA). Briefly, aliquots of standard controls and samples of plasma from the study subjects (in triplicate) were loaded (10 µl each) onto a 96-microwell plate pre-coated with a polyclonal antibody raised against cotinine. After adding a cotinine horseradish peroxidase enzyme (100 µl per well), the microplate was incubated for one hour at room temperature in the dark. Unbound cotinine and cotinine enzyme-conjugate were washed off by rinsing the wells six times with distilled water (300 µl each wash). A chromogenic substrate (3,3',5,5'-Tetramethylbenzidine) was added (100 µl per well), and the plate was incubated for 30 min at room temperature. The reaction was terminated by adding a stop solution (100 µl per well), and absorbance was read at 450 nm using a SpectraMax i3x Multi-Mode Detection Platform (Molecular Devices, LLC., San Jose, CA). Results are expressed as nanograms (ng) of cotinine measured per milliliter of plasma^15^.

### Statistics

Results are expressed as median ± SE in the text. Comparisons of all variables between two groups were performed by the Wilcoxon Rank-Sum test. Relationships between different variables were examined by Spearman’s rank correlation analysis. Applied tests for other analyses are specified in the text. All statistical tests were two-sided. *P* values < 0.05 were considered statistically significant. All statistical analyses were performed using the R environment for statistical computing, available at RStudio (https://rstudio.com/), which is a free and open-source software. Related statistical tests for functional analysis are incorporated in IPA (www.ingenuity.com). For power analysis, we used the package ssizeRNA^66^, which is designed to provide an estimation of sample size while controlling FDR^21^ for RNA-seq experimental design. The method approximates the average power across the differentially expressed genes, and then calculates the sample size to achieve a desired average power, while controlling FDR^66^. The method can also be used for post hoc power analysis^66^. The manual page for the check.power function can be found at: https://rdrr.io/cran/ssizeRNA/man/check.power.html. Raw code for the check.power function can be found on the public github for the CRAN ssizeRNA package at: https://github.com/cran/ssizeRNA/blob/master/R/check.power.R. We performed post hoc power analysis using the check.power function^66^, which computes the observed power and true FDR by Benjamini and Hochberg’s method^21^, based on our sample size of 37 for vapers, 22 for smokers, and 23 for controls. The results were based on 100 simulations, confirming that the calculated power was achieved while FDR was controlled successfully.

## Supplementary Information

Supplementary Legends.

Supplementary Figure 1.

Supplementary Figure 2.

Supplementary Table S1.

Supplementary Table S2.

## Data Availability

The raw RNA-seq data have been deposited in the Gene Expression Omnibus database at NCBI (https://www.ncbi.nlm.nih.gov/geo/), and accession number is GSE169757.

## Abbreviations

∆Ψ_m_: Mitochondrial membrane potential
8-OHdG: 8-Hydroxy-2'-deoxyguanosine
ACTB: Actin beta
BMI: Body mass index
CCR5: C–C Motif Chemokine Receptor 5
COPD: Chronic obstructive pulmonary disease
cpm: Count per million
cum e-liq: Cumulative e-liquid
DAMPs: Damage-associated molecular patterns
DE: Differential expression
DEGs: Differentially expressed genes
e-cigs: Electronic cigarettes
ELISA: Enzyme-linked immunosorbent assay
FC: Fold change
FDR: False discovery rate
GAPDH: Glyceraldehyde-3-phosphate dehydrogenase
IFN: Interferon
IL: Interleukin
IPA: Ingenuity Pathway Analysis
lncRNAs: Long non-coding RNAs
MT-rRNAs: Mitochondrial ribosomal RNAs
MT-tRNAs: Mitochondrial transfer RNAs
ncRNAs: Non-coding RNAs
OXPHOS: Oxidative phosphorylation
PY: Pack year
RNA-seq: RNA-sequencing
RT-qPCR: Reverse-transcription quantitative polymerase chain reaction
ROS: Reactive oxygen species
TMM: Trimmed mean of M-values
