# Supplementary material for: A novel role for vaping in mitochondrial gene dysregulation and inflammation fundamental to disease development
Source: Sci Rep. 2021 Nov 23;11:22773. doi: 10.1038/s41598-021-01965-1 (PMC8611078; doi:10.1038/s41598-021-01965-1)
Supplement: Supplementary file 1 — Supplementary Legends. [file 41598_2021_1965_MOESM1_ESM.pdf]

A novel role for vaping in mitochondrial gene dysregulation and inflammation fundamental to disease development. Tommasi *et al*.

"Legend for Supplementary Figures"

Figure S1. Top Diseases and BioFunctions analysis of differentially expressed genes in vapers and smokers by IPA. The heat map shows the top diseases and biological functions associated with the differentially expressed genes in vapers and smokers. Orange and blue squares indicate the predicted increase or decrease of the disease/biofunction, respectively.

Figure S2. Validation of RNA-seq expression data by RT-qPCR. Transcription levels of randomly selected up- and down-regulated genes in vapers and smokers relative to controls, as determined by RNA-seq, were validated by RT-qPCR. Normalized read counts for each target gene determined by RNA-seq, were correlated to median normalized expression levels of the corresponding gene quantified by RT-qPCR, using the Spearman Rank correlation analysis.
