# Supplementary figures and images for: A novel role for vaping in mitochondrial gene dysregulation and inflammation fundamental to disease development
Source: Sci Rep. 2021 Nov 23;11:22773. doi: 10.1038/s41598-021-01965-1 (PMC8611078; doi:10.1038/s41598-021-01965-1)

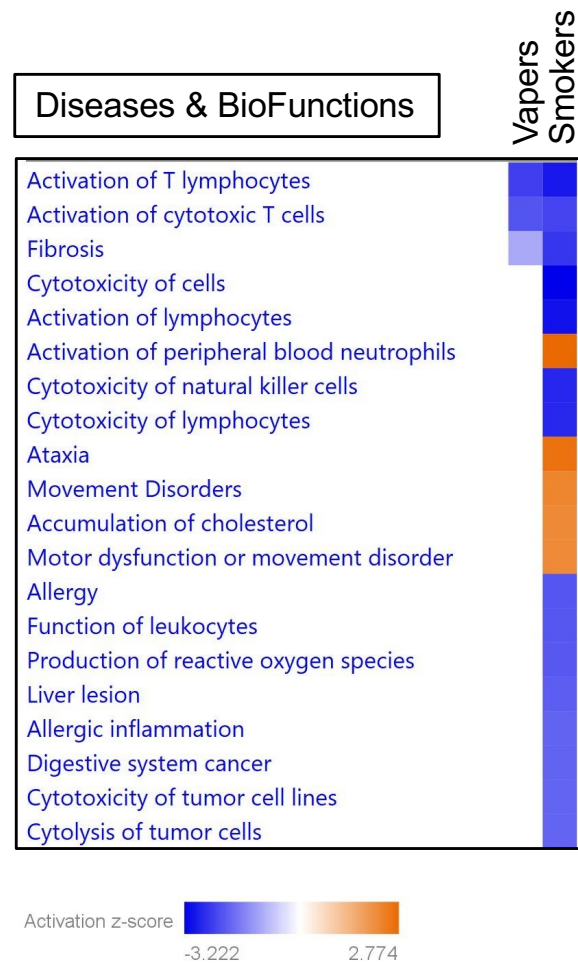

Supplement: Supplementary file 2 — Supplementary Figure 1. [file 41598_2021_1965_MOESM2_ESM.pdf]

### PRSS23

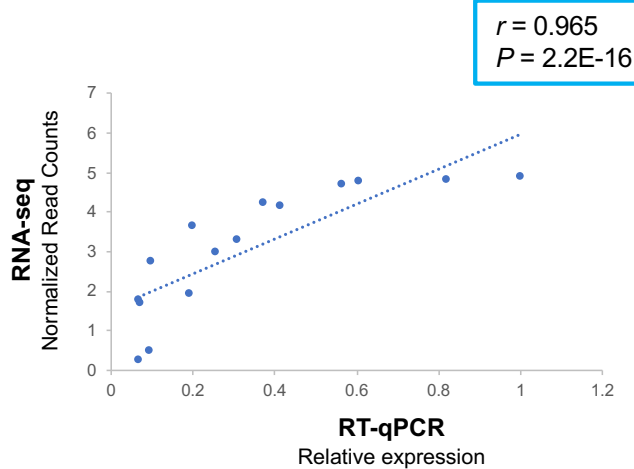

### SLC1A7

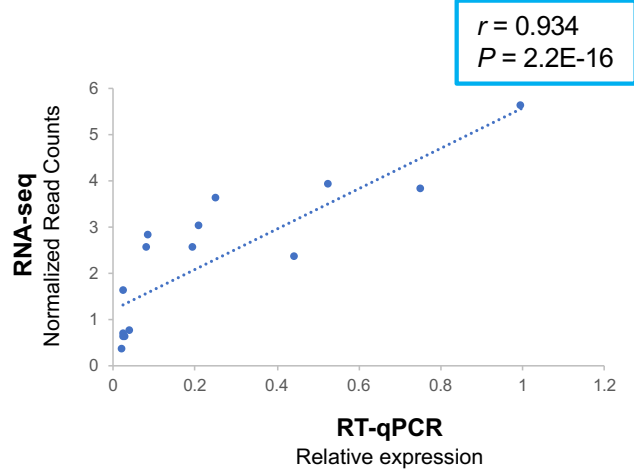

### B3GAT1

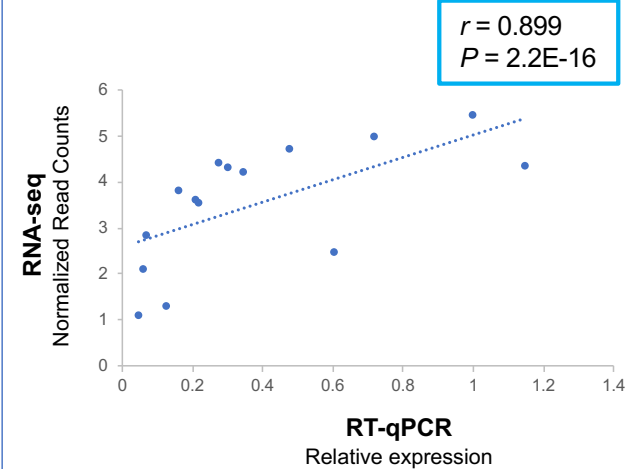

### COL27A1

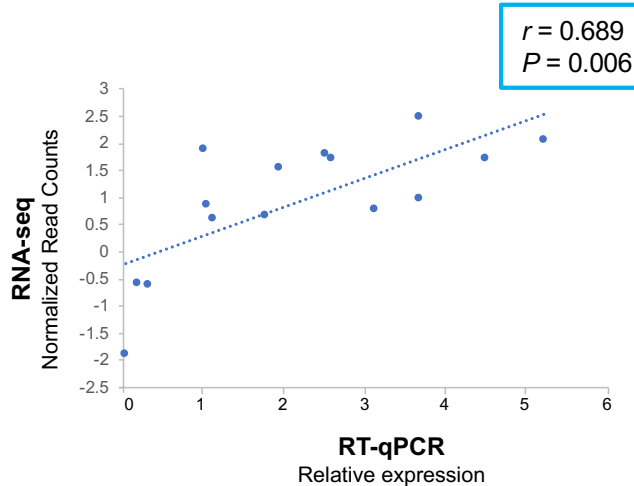

### NUAK1

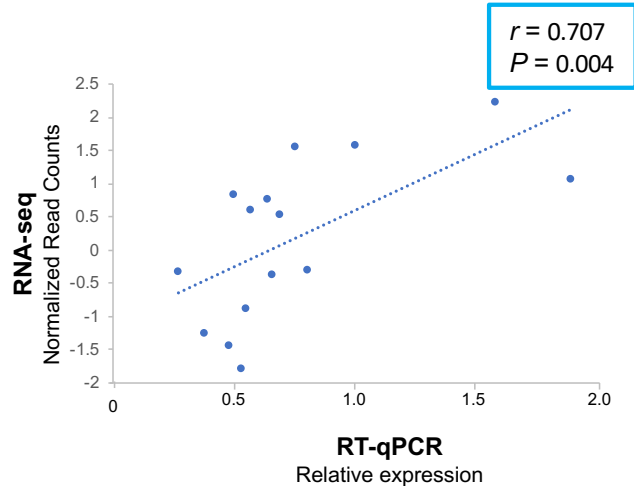

### NINL

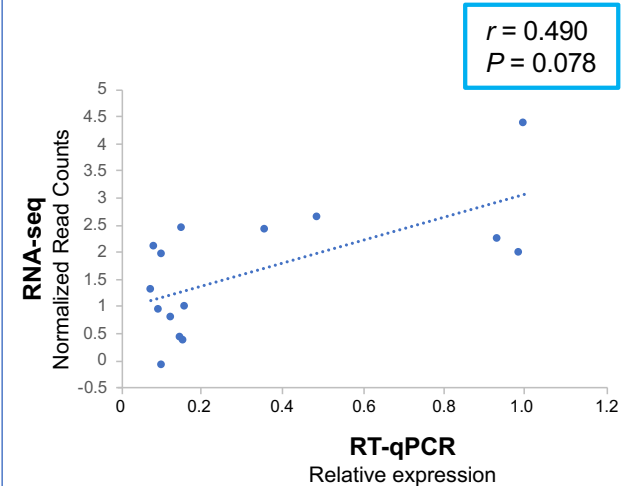

Supplement: Supplementary file 3 — Supplementary Figure 2. [file 41598_2021_1965_MOESM3_ESM.pdf]
